# Peripheral immunity and risk of incident brain disorders: a prospective cohort study of 161,968 participants

**DOI:** 10.1038/s41398-023-02683-0

**Published:** 2023-12-09

**Authors:** Xiaoling Zhong, Yixuan Qiang, Ling Wang, Yaru Zhang, Jieqiong Li, Jianfeng Feng, Wei Cheng, Lan Tan, Jintai Yu

**Affiliations:** 1 Department of Neurology, Qingdao Municipal Hospital, Qingdao University, Qingdao, China; 2 Department of Neurology, Affiliated Qingdao Central Hospital of Qingdao University, Qingdao Cancer Hospital, Qingdao, China; 3 Department of Neurology and Institute of Neurology, Huashan Hospital, State Key Laboratory of Medical Neurobiology and MOE Frontiers Center for Brain Science, Shanghai Medical College, Fudan University, National Center for Neurological diseases, Shanghai, China; 4 Department of Neurology, the Affiliated Hospital of Qingdao University, Qingdao, Shandong China; 5 The Institute of Science and Technology for Brain-Inspired Intelligence, Fudan University, Shanghai, China

**Keywords:** Predictive markers, Learning and memory

## Abstract

Whether peripheral immunity prospectively influences brain health remains controversial. This study aims to investigate the longitudinal associations between peripheral immunity markers with incident brain disorders. A total of 161,968 eligible participants from the UK Biobank were included. We investigated the linear and non-linear effects of peripheral immunity markers including differential leukocytes counts, their derived ratios and C-reactive protein (CRP) on the risk of dementia, Parkinson’s disease (PD), stroke, schizophrenia, bipolar affective disorder (BPAD), major depressive disorder (MDD) and anxiety, using Cox proportional hazard models and restricted cubic spline models. Linear regression models were used to explore potential mechanisms driven by brain structures. During a median follow-up of 9.66 years, 16,241 participants developed brain disorders. Individuals with elevated innate immunity markers including neutrophils, monocytes, platelets, neutrophil-to-lymphocyte ratio (NLR), and systemic immune-inflammation index (SII) had an increased risk of brain disorders. Among these markers, neutrophils exhibited the most significant correlation with risk of dementia (hazard ratio 1.08, 95% confidence interval 1.04–1.12), stroke (HR 1.06, 95% CI 1.03–1.09), MDD (HR 1.13, 95% CI 1.10–1.16) and anxiety (HR 1.07, 95% CI 1.04–1.10). Subgroup analysis revealed age-specific and sex-specific associations between innate immunity markers with risk of dementia and MDD. Neuroimaging analysis highlighted the associations between peripheral immunity markers and alterations in multiple cortical, subcortical regions and white matter tracts, typically implicated in dementia and psychiatric disorders. These findings support the hypothesis that neuroinflammation is important to the etiology of various brain disorders, offering new insights into their potential therapeutic approaches.

## Introduction

Neurological and psychiatric disorders are a group of chronic diseases that often cause impaired cognition, motor function, emotional regulation, and tactile function [1]. These disorders include neurological conditions like dementia, PD and stroke, as well as psychiatric conditions such as schizophrenia, BPAD, MDD and anxiety. and are the leading causes of global mortality and disability [2]. Neurological and psychiatric disorders can be caused by external stresses and internal genetic factors. Multiple modifiable factors involving various aspects of life have been identified as essential for the prevention of brain disorders. These factors mainly encompass maintaining healthy lifestyle habits and management of risk factors, which includes regular exercise, normal social interactions, balanced nutrition, quality sleep, avoidance of toxins, alcohol, and smoking, as well as effectively managing medical risk factors such as hypertension, hyperlipidemia, and diabetes [1]. As such, unraveling the underlying risk factors may provide insights into disease mechanisms and find potential therapeutic targets.

The immune system has a critical role in brain homeostasis, resilience and brain reserve, and the interaction between the immune system and brain is garnering growing interest across a broad range of neurological and psychiatric disorders [3–5]. The peripheral immune system comprises innate and adaptive immune systems. The components of the innate immune response mainly include monocytes, macrophages, neutrophils, dendritic cells, mast cells, and natural killer cells [6, 7]. On the other hand, the adaptive immune response involves B cells and T cells [7]. An effective way to assess peripheral immunity is by analyzing differential leukocyte counts obtained from peripheral blood. Moreover, ratios of leukocyte counts, including NLR, platelet-to-lymphocyte ratio (PLR), LMR, and SII, have been proposed as more effective measures to assess the strength of peripheral immunity [8]. CRP is a classical acute-phase reactant protein from the pentraxin family. It can remain elevated in chronic inflammatory conditions, and thus could be regarded as a marker of peripheral immunity [9]. Emerging evidence has shown the association between peripheral immunity and risk of dementia [10–15], PD [16–22], stroke [23], as well as schizophrenia [24–27], BPAD [28–30], and MDD [31, 32]. However, most studies focus on the association between CRP and incident brain disorders, there is limited and inconsistent evidence concerning the association between differential leukocytes and incident brain disorders. Furthermore, previous studies have been limited by relatively small sample sizes, short durations of follow-up, or insufficient statistical power to identify clinically significant associations.

In this study, we conducted a comprehensive analysis using data from the UK Biobank (UKB), a large prospective cohort study. We chose nine peripheral immunity markers which could represent peripheral immune status and readily accessible from peripheral blood. We aimed to investigate the relationships between these markers and the risk of seven incident brain disorders, including dementia, PD, stroke, schizophrenia, BPAD, MDD, and anxiety.

## Methods

### Participants

UKB is a large-scale longitudinal cohort database containing in-depth genetic and health information of 502,493 participants aged 37–73 years at baseline assessments recruited from 22 centers throughout the UK during 2006–2010 (https://www.ukbiobank.ac.uk/). Data covering lifestyles and health conditions, physical measures, biological samples, imaging, and genotyping were obtained from interviews, questionnaires and measurements [33]. The baseline assessment was followed by consecutive long-term follow-up. The database is linked to national health datasets, including primary care, hospital inpatient, death, and cancer registration data [33]. Ethics approval for the UKB study was obtained from the North West Multicenter Research Ethical Committee. All participants provided written informed consent. The present study was conducted under UKB application number 19542. Participants with baseline diagnoses of the seven brain disorders, with a follow-up duration of less than five years or below 55 years, were excluded from our study. Additionally, we excluded individuals with conditions that could impact peripheral immunity markers, including malignant neoplasms, blood and blood-forming organ diseases, autoimmune diseases, and chronic inflammatory conditions. Finally, 161,968 participants were included in the primary analysis.

### Peripheral immunity markers

Blood samples were analyzed at the UKB central laboratory within 24 h of the blood draw. Peripheral blood cell counts were obtained from an automated, clinically validated Coulter LH 750. Quality control was carried out according to the manufacturer’s recommendations. More details were available in the UK Biobank online showcase and protocol (http://www.ukbiobank.ac.uk). Thirty-one parameters were reported by the instrument (details available in https://biobank.ndph.ox.ac.uk/showcase/ukb/docs/ haematology.pdf). Baseline count data of neutrophils, monocytes, platelets, and lymphocytes were extracted. Next, we calculated four ratios based on peripheral blood cell counts, including NLR (neutrophils/lymphocytes), PLR (platelets/lymphocytes), SII (neutrophil×platelets/lymphocytes), and LMR (lymphocytes/monocytes). In addition, the serum CRP concentration was measured by immunoturbidimetric-high sensitivity analysis on Beckman Coulter AU5800 at baseline, ranging from 0.08 to 79.96 mg/L. Peripheral immunity markers with corresponding data fields are listed in Table S1 in Additional files 1.

### Covariates

Relevant covariates were measured at baseline (corresponding field ID was presented in Table S1 in Additional files 1). Demographic variables included age (years), sex (male or female), and ethnicity (white or non-white). Education was categorized as higher (college/university degree or other professional qualification) or lower. Systolic blood pressure (SBP) and diastolic blood pressure (DBP) were measured twice at an interval time for 5 min or more using IntelliSense blood pressure monitor model HEM-907XL (Omron) [34]. We performed the test for multi-collinearity of covariates and variance inflation factor (VIF) < 10 indicated no multi-colinearities (Table S2 in Additional files 1).

### Brain disorders

The outcomes included seven brain disorders, namely neurological disorders (dementia, PD, and stroke) and psychiatric disorders (schizophrenia, BPAD, MDD, and anxiety). The brain disorders were verified and classified according to the corresponding three-character ICD-10 codes, extracted from first occurrences of health outcomes (Category 1712). The health outcomes were generated from self-report conditions, primary care records, hospital inpatient data, and death records obtained from the National Health Service (NHS). The UK Biobank website [35] provides further details about the data processing and linkage procedures [35]. All-cause dementia encompassed codes F00, F01, F02, F03, and G30, PD was identified by code G20, stroke by codes I60-I69, MDD by codes F32 and F33, schizophrenia by code F20.9, BPAD by code F31 and anxiety by codes F40 and F41. Follow-up duration was calculated from the date of the initial assessment (Field 53) to the earliest date of any incident brain disorder diagnosis, date of death (Field 40000), or the last available date extracted from hospital inpatient data (Field 41280-41281) or recorded by the general practitioners (Field 42040), whichever came first.

### Brain imaging data

Brain magnetic resonance imaging (MRI) data from over 40,000 participants in the UK Biobank was collected using a standardized protocol on a 3 T Siemens Skyra scanner with a 32-channel head coil. The T1-weighted images were processed by the UK Biobank imaging team using FSL software. Various imaging-derived phenotypes (IDPs) were generated, including cortical volume, area and thickness, subcortical volume, fractional anisotropy (FA) and mean diffusivity (MD) measures. We included participants with peripheral immunity and MRI data, and excluded those with major brain disorders. In total, 40068 participants were included. The volume, area and thickness of 68 cortical regions, volume of 45 subcortical regions, the FA and MD values of the 48 white matter tracts were analyzed to investigate the associations between brain structure and peripheral immunity markers. Further details of imaging processing and quality control can be found in the provided open-access article [36].

### Statistical analysis

The results for continuous variables are shown as mean and standard deviation (SD) (normal distribution), while categorical variables are shown as number and percentage. To compare the effect sizes between various exposures, the peripheral immunity markers were log-transformed and standardized to *Z* scores (*Z* = (value − mean)/SD). Hence, the hazard ratio (HR) reflects the predicted effect of per SD increment of the peripheral immunity markers. We assessed longitudinal associations between various peripheral immunity markers and the risk of seven brain disorders with multivariable Cox proportional hazard regression models. The number of days between the baseline visit and dementia occurrence was calculated and utilized for Cox regression analysis. Covariates including age, sex, ethnicity, education, SBP and DBP were adjusted. The P values were further corrected by false-discovery rate (FDR) (labeled as Q values) to avoid the inflation of false-positive results. Restricted cubic spline (RCS) analysis was further performed to explore the potential non-linear relationships between peripheral immunity markers and the risk of various brain disorders. The spline models were adjusted with the same set of covariates as in the Cox model. To assess the robustness of our findings, we further performed two sensitivity analyses: (1) exclusion of participants with the extreme values (>mean ± 3 SD) of all exposures, (2) imputing missing values for all relevant exposures and covariates using CART, as implemented in the ‘mice’ package in R (single imputation, seed = 123). In subgroup analyses, we explored differences for each exposure based on age (55–65 years, ≥65 years) at baseline assessment and sex (male, female). Finally, multiple linear regression models were used to explore the associations between peripheral immunity markers and brain morphometric measures after adjusting for the aforementioned covariates. All analyses were carried out using R version 4.0.2.

## Results

### Population characteristics

**Fig. 1:**
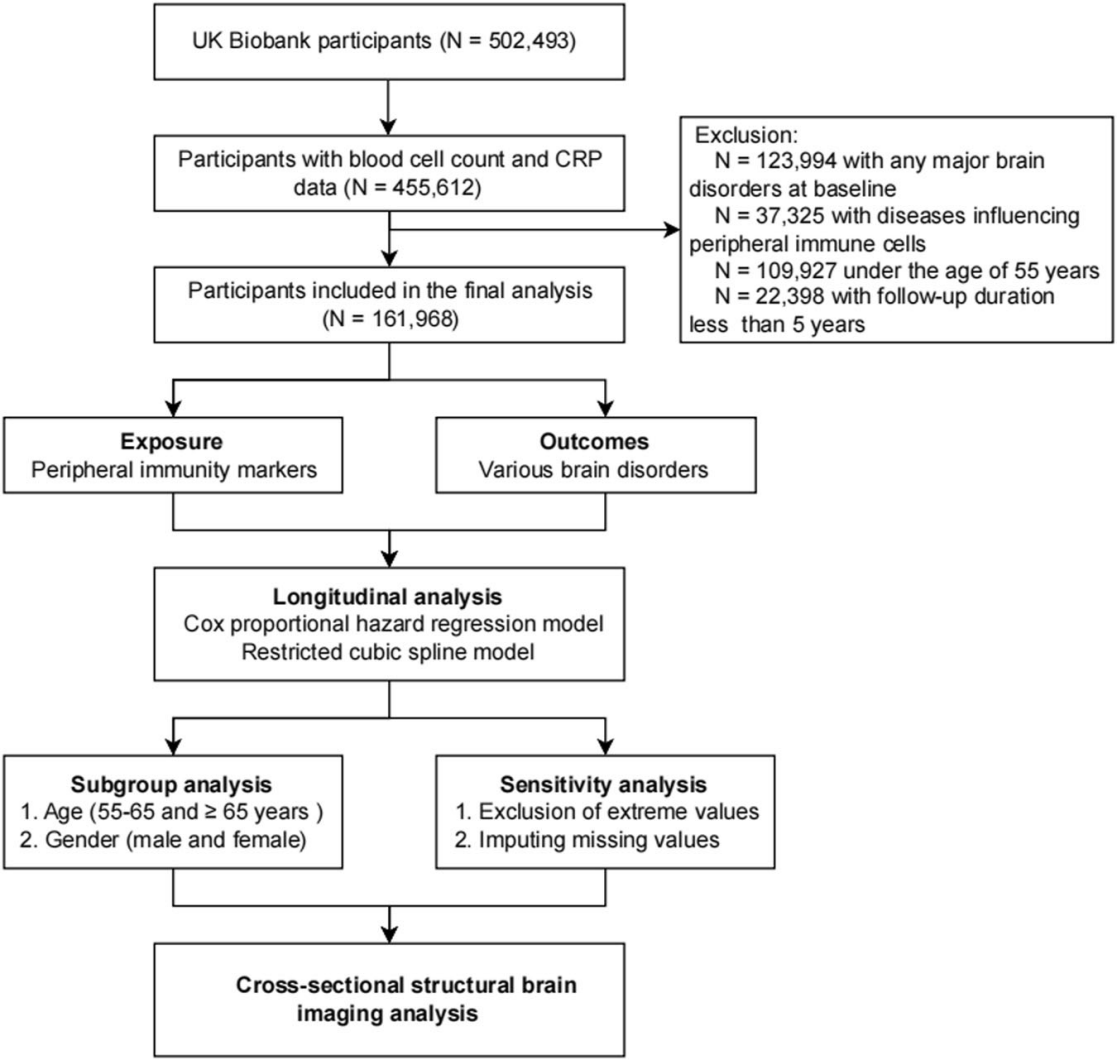
Flow chart of study design. Five hundred and two thousand four hundred and ninety-three participants aged 37–73 years were included for initial assessment, after excluding participants with baseline diagnoses of the seven brain disorders, with a follow-up duration of less than five years or below 55 years, with conditions that could impact peripheral immunity markers. Finally, 161,968 participants were included in the primary analysis.

In this study, 161,968 participants were included, with an average age of 62.14 (SD = 4.07) years and 50.7% of them were women. A flow chart of study design was shown in Fig. 1. During a median follow-up of 9.66 years, 2958 individuals were diagnosed with dementia, 1193 with PD, 4815 with stroke, 51 with schizophrenia, 63 with BPAD, 3786 with MDD, and 4691 with anxiety. Table 1 presents the participants’ baseline demographic characteristics and peripheral immunity markers regarding brain health status. Compared to the control group, participants in the incident brain disorder group had characteristics such as older age, lower education levels, higher systolic blood pressure, and higher levels of innate immunity markers (Table 1).

**Table 1:** Baseline characteristics of UKB participants by brain health status.

Characteristics	Overall (*N* = 161,968)	No incident brain disorder (*N* = 145,727)	Incident brain disorder (*N* = 16,241)	*P* value
Age (mean (SD))	62.14 (4.07)	62.02 (4.05)	63.28 (4.05)	<0.001
Sex (%)				
Male	79816 (49.3)	71,921 (49.4)	7895 (48.6)	0.074
Female	82,152 (50.7)	73,806 (50.6)	8346 (51.4)	
Education (%)				
Low	88,169 (55.2)	78,587 (54.6)	9582 (60.3)	<0.001
High	71,605 (44.8)	65,284 (45.4)	6321 (39.7)	
Ethnicity				
Non-white	5544 (3.4)	5022 (3.5)	522 (3.2)	0.132
White	1,55,756 (96.6)	1,40,112 (96.5)	15,644 (96.8)	
SBP (mean (SD))	142.49 (18.59)	142.36 (18.53)	143.62 (19.02)	<0.001
DBP (mean (SD))	82.71 (9.93)	82.73 (9.91)	82.57 (10.12)	0.074
Lymphocyte count (mean (SD))	1.97 (1.04)	1.97 (1.05)	1.98 (1.03)	0.256
Monocyte count (mean (SD))	0.49 (0.21)	0.48 (0.21)	0.49 (0.20)	<0.001
Neutrophil count (mean (SD))	4.20 (1.34)	4.18 (1.34)	4.32 (1.39)	<0.001
Platelet count (mean (SD))	249.07 (58.46)	248.89 (58.19)	250.63 (60.81)	<0.001
CRP (mean (SD))	2.62 (4.27)	2.60 (4.24)	2.79 (4.57)	<0.001
NLR (mean (SD))	2.34 (1.24)	2.33 (1.23)	2.42 (1.34)	<0.001
LMR (mean (SD))	4.54 (4.13)	4.55 (4.20)	4.49 (3.44)	0.078
PLR (mean (SD))	139.28 (81.08)	139.16 (82.80)	140.38 (63.67)	0.069
SII (mean (SD))	583.69 (358.02)	580.99 (352.21)	607.91 (405.63)	<0.001

Data presented as mean (SD) for continuous variables and number (%) for categorical variables. Education level was categorized as higher (college/university degree or other professional qualification) or lower.

*SBP* systolic blood pressure, *DBP* diastolic blood pressure, *CRP* C-reactive protein, *NLR* neutrophils/lymphocytes ratio, *LMR* lymphocytes/monocytes ratio, *PLR* platelets/lymphocytes ratio, *SII* systemic immune-inflammation index (neutrophils × platelets/lymphocytes).

### Peripheral immunity markers and risk of brain disorders

**Fig. 2:**
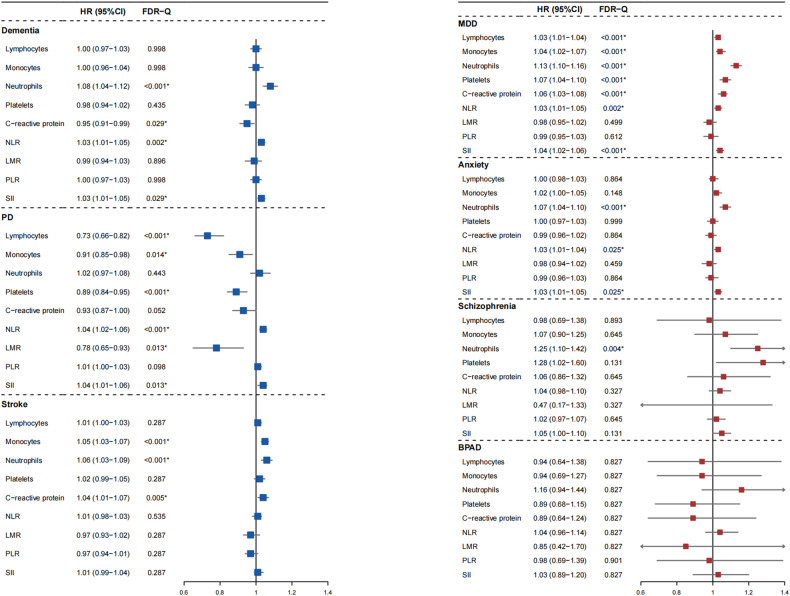
Linear associations between peripheral immunity markers and incident brain disorders. The model was adjusted for age at baseline, sex, ethnicity, education, SBP and DBP. Exposures were log-transformed and standardized to *Z* score so that the HR represents the predicted effect of a one SD increment. Statistical significance at FDR-adjusted *P* (labeled as Q values) < 0.05. FDR, false discovery rate; HR hazard ratio, SD standard deviation, SBP systolic blood pressure, DBP diastolic blood pressure, PD Parkinson’s disease, MDD major depressive disorder, BPAD bipolar affective disorder.

We investigated the relationship between nine peripheral immunity markers and the incidence of seven brain disorders with the Cox model by adjusting for age at baseline, sex, ethnicity, education, SBP and DBP. Several markers, especially innate immunity markers, were associated with the risk of various brain disorders (Fig. 2).

**Fig. 3:**
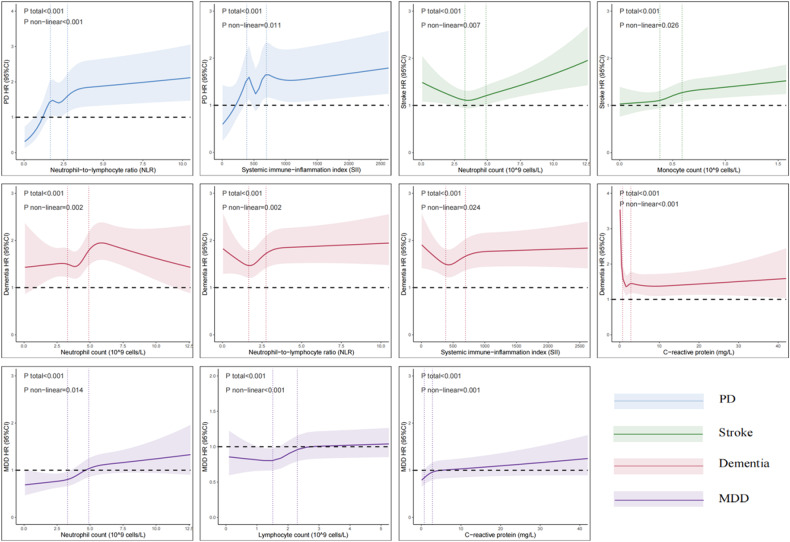
Non-linear associations between peripheral immunity markers and incident brain disorder. Restricted cubic spline models fitted for Cox proportional hazards models was utilized, and 11 significant non-linear associations were identified. Two dashed vertical lines indicate 25% and 75% values of each exposure. Results were adjusted for age at baseline, sex, ethnicity, education, SBP and DBP. The colors blue, green, red, and purple represent each brain disorder fitting into PD, stroke, dementia, and MDD categories. HR hazard ratio, PD Parkinson’s disease, MDD major depressive disorder.

In innate immunity category, higher neutrophils, the main element of innate immunity, were significantly associated with increased risk of incident dementia (HR 1.08, 95% CI: 1.04–1.12, *p* < 0.001), stroke (HR 1.06, 95% CI: 1.03–1.09, *p* < 0.001), MDD (HR, 1.13 95% CI: 1.10–1.16, *p* < 0.001), and anxiety (HR 1.07, 95% CI: 1.04–1.10, *p* < 0.001). In addition, a non-linear relationship was found between neutrophils and risk of incident dementia (*p* for non-linearity = 0.002; Fig. 3), stroke (*p* for non-linearity = 0.007; Fig. 3) and MDD (*p* for non-linearity = 0.014; Fig. 3). Elevated levels of NLR and SII, ratios of innate immunity to adaptive immunity, were observed to have similar results. Specifically, they were associated with an increased risk of dementia (HR 1.03, 95% CI: 1.01–1.05, *p* = 0.002 and HR 1.03, 95% CI: 1.01–1.05, *p* = 0.029, respectively), PD (HR 1.04, 95% CI: 1.02–1.06, *p* < 0.001 and HR 1.04, 95% CI: 1.01–1.06, *p* = 0.013, respectively), MDD (HR 1.03, 95% CI: 1.01–1.05, *p* = 0.002 and HR 1.04, 95% CI: 1.02–1.06, *p* < 0.001, respectively), and anxiety (HR 1.03, 95% CI: 1.01–1.04, *p* = 0.025 and HR 1.03, 95% CI: 1.01–1.05, *p* = 0.025, respectively). Additionally, the RCS model revealed a statistically significant non-linear relationship between NLR (*p* for non-linearity = 0.002, *p* for non-linearity < 0.001; Fig. 3) and SII (*p* for non-linearity = 0.024, *p* for non-linearity = 0.011; Fig. 3), with the risk of both dementia and PD. High monocyte count was found to be associated with an increased risk of stroke (HR 1.05, 95% CI: 1.03–1.07, *p* < 0.001), MDD (HR 1.04, 95% CI: 1.02–1.07, *p* < 0.001), and decreased risk of PD (HR 0.91, 95% CI: 0.85–0.98, *p* = 0.014). Levels of CRP were found to be associated with the risk of incident dementia (HR 0.95, 95% CI: 0.91–0.99, *p* = 0.029), stroke (HR 1.04, 95% CI: 1.01–1.07, *p* = 0.005) and MDD (HR 1.06, 95% CI: 1.03–1.08, *p* < 0.001). Moreover, a significant non-linear relationship between CRP and risk of dementia (*p* for non-linearity < 0.001; Fig. 3) and MDD (*p* for non-linearity = 0.001; Fig. 3) was observed. Low platelet count was associated with an increased risk of incident PD (HR 0.89, 95% CI: 0.84–0.95, *p* < 0.001), while higher platelet count was associated with an increased risk of incident MDD (HR 1.07, 95% CI: 1.04–1.10, *p* < 0.001).

In the adaptive immunity category, increased adaptive immunity markers were associated with decreased risk of PD (HR 0.73, 95% CI: 0.66–0.82, *p* < 0.001 for lymphocytes and HR 0.78, 95% CI: 0.65–0.93, *p* = 0.013 for LMR) but increased risk of MDD (HR 1.03, 95% CI: 1.01–1.04, *p* < 0.001 for lymphocytes).

### Sensitivity and subgroup analysis

Sensitivity analysis excluding the extreme values or imputing missing values did not significantly change the risk estimates. In subgroup analysis, HRs were generally in the same direction between peripheral immunity markers and multiple brain disorders (Table S3 in Additional files 1). In age-stratified subgroups, the associations between peripheral immunity markers and the risk of PD, stroke, anxiety, and BPAD incidence were consistent with the general population (Table S4 in Additional files 1). Interestingly, the association between peripheral immunity markers and MDD risk was significant only in participants aged 55 to 65, whereas the relationship between peripheral immunity markers and dementia risk was more pronounced in participants over 65 years (Table S4). In sex-stratified subgroups, the associations were generally more prominent in males than in females. Notably, significant associations were found between peripheral immunity markers and the risk of incident dementia exclusively in males (Table S5 in Additional files 1).

### Association between peripheral immunity markers and brain structure

**Fig. 4:**
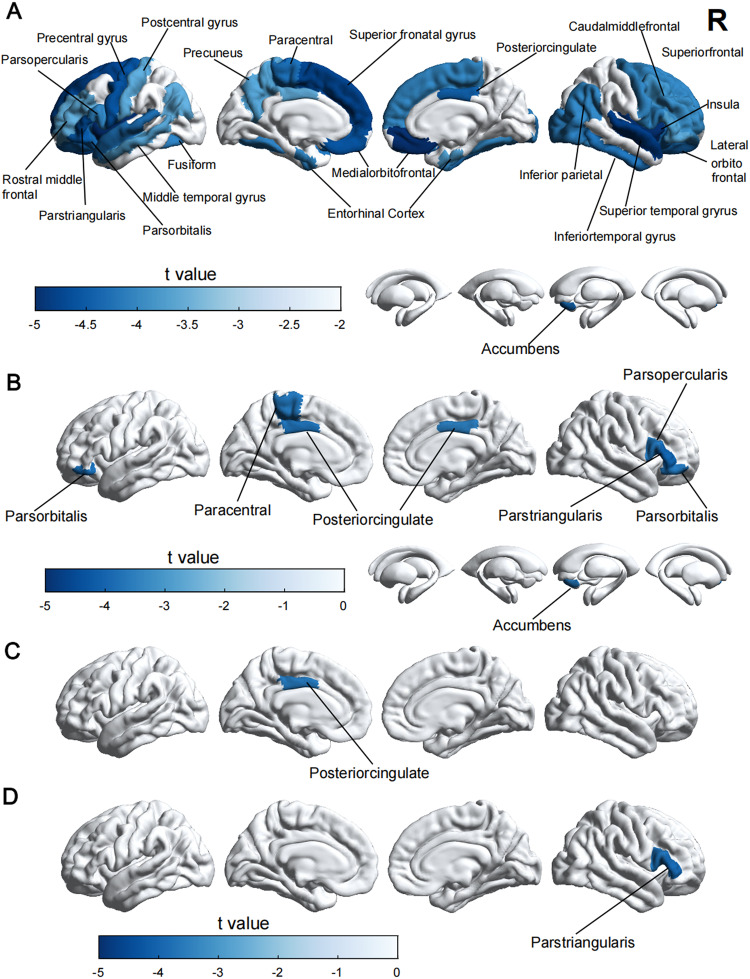
Associations between peripheral immunity markers and brain structure. Neutrophil (**A**), SII (**B**), platelet (**C**), NLR (**D**) are negatively associated with volume of cortical and subcortical structures (Bonferroni-corrected, *P* < 0.05). Models are adjusted for age at baseline, sex, ethnicity, education, SBP and DBP. *T*-value represents the correlation coefficient of the linear regression. SBP systolic blood pressure, DBP diastolic blood pressure, NLR neutrophils/lymphocytes ratio, SII systemic immune-inflammation index (neutrophils×platelets/lymphocytes).

After FDR correction, results showed significant associations between innate immunity markers and multiple brain regions. Notably, neutrophils was associated with volumes of the insula (left hemisphere, *P* < 0.001, right hemisphere, *P* < 0.001), medial orbital frontal cortex (left hemisphere, *P* = 0.001, right hemisphere, *P* < 0.001), superior temporal gyrus (left hemisphere, *P* = 0.003, right hemisphere, *P* < 0.001), pars triangularis (left hemisphere, *P* < 0.001, right hemisphere, *P* = 0.022), precentral gyrus (left hemisphere, *P* < 0.001, right hemisphere, *P* < 0.006), posterior cingulate gyrus (left hemisphere, *P* = 0.034, right hemisphere, *P* = 0.001), as well as area and thickness of several cortical regions and multiple white matter tracts (Fig. 4; Tables S6–10 in Additional files 1).

Similarly, significant associations were identified between other innate immunity markers, including platelet, NLR and SII, with multiple cortical and subcortical regions implicated in dementia and psychiatric disorders (Fig. 4; Tables S8–10 in Additional files 1). In addition, these markers were observed to correlate with FA or MD values of white matter tracts, such as anterior corona radiate, anterior limb of internal capsule, cerebral peduncle, superior fronto-occipital fasciculus, and cingulum cingulate gyrus (Tables S6–7 in Additional files 1).

## Discussion

Utilizing data from 161,968 participants in the UKB, we explored the associations between nine peripheral immunity markers and the risk of seven incident brain disorders. Our study yielded several noteworthy findings. Firstly, innate immunity markers including neutrophils, NLR, SII and CRP exhibited significant correlations with risk of various brain disorders, including dementia, PD, stroke, MDD, and anxiety. Secondly, multiple non-linear relationships were observed between peripheral immunity markers and incident brain disorders. Thirdly, subgroup analysis revealed that the relationship between peripheral immunity markers and risk of incident dementia is more pronounced in males and older individuals, whereas the relationship with the risk of incident MDD is more prominent among younger individuals. Lastly, neuroimaging analysis revealed the associations between peripheral immunity markers and alterations in multiple cortical, subcortical regions and white matter tracts, typically implicated in dementia and psychiatric disorders.

Our findings indicate that abnormal innate immunity is not specific to a particular disorder but could reflect an underlying pathological process that leads to brain dysfunction. The mechanisms by which innate immunity contributes to brain disorders are complicated. Under normal physiological conditions, leukocytes do not easily penetrate the blood-brain barrier (BBB) and are predominantly found near brain boundaries. On the flip side, peripheral cytokines can penetrate the brain via endothelial transporters, enabling them to impact brain functions by modulating neurotransmitters and neural activity [37]. In cases of chronic inflammation, the integrity of the BBB can be disrupted, facilitating the entry of circulating cytokines and leukocytes into the brain [38]. Evidence has shown that the peripheral neutrophils could release neutrophil extracellular traps (NETs) and contribute to the development and aggravation of lipopolysaccharide (LPS)-induced depressive behaviors in mice [39]. In transgenic models of AD, it has been observed that neutrophils extravasate from the bloodstream and accumulate in regions where amyloid-β (Aβ) deposits are present [40]. In individuals with AD, neutrophils have been observed to adhere to and intrude into brain venules and be present in the parenchyma [40]. Once peripheral inflammatory signals flow into the brain, microglia, the primary resident immune cells can be activated [41], thereby contributing to chronic neuroinflammation and neurodegeneration. Moreover, neutrophils also actively promote all stages of atherosclerosis by facilitating the recruitment of monocytes, activating macrophages, and exerting cytotoxic effects [42]. CRP could induce direct neuronal damage through pro-inflammatory responses [43], and act as a cardiovascular risk factor leading to the development of cerebral atherosclerosis [44, 45]. The gut-liver-brain axis has drawing much attention in exploring the association between peripheral immunity and brain disorders. Chronic gastrointestinal inflammation could lead to increased intestinal permeability, which is known as a leaky gut syndrome (LGS) [46]. The disruption of the intestinal barrier allows the leak of antigens from the digestive system into the bloodstream through the portal vein, subsequently activating liver immunity [47]. Meanwhile, systemic inflammation triggered by LGS could cause increased pro-inflammatory cytokines level, the excess of which is destructive to host cells, including cells of the CNS [48]. Peripheral immunity is also implicated in the relationship between psychosocial stress and neuropsychiatric diseases. There is now evidence that in experimental animals, different types of psychosocial stressors increase systemic and CNS levels of pro-inflammatory cytokines, including IL-1 and IL-6 [49, 50]. Moreover, in humans, psychosocial stress induced increase in pro-inflammatory cytokines coordinate stress-induced changes in peripheral blood immune cells, sparking inflammatory reactions and neurobehavioral changes [51, 52]. These may be the mechanistic explanations for the significant associations between innate immune dysfunction and the risk of various brain disorders, including dementia, stroke, depression and anxiety in our study.

As for neurodegenerative disorders, although many studies have identified changes in peripheral immunity markers in individuals with dementia [53–58], limited cohort studies have investigated the association between peripheral immunity markers and dementia incidence prospectively [10, 59, 60]. Several observational studies have evaluated the associations between plasma CRP levels and risk of dementia, and the results were inconclusive [12, 54, 61–71]. Our study, for the first time, investigated and discovered a non-linear relationship between CRP levels and incident dementia. Interestingly, contradictory results were yielded from Cox and RCS analysis regarding the association between CRP and risk of dementia. The reason may lie in the complex non-linear relationships between CRP levels and incident dementia, which cannot be captured adequately by a linear model. Consequently, the linear results may introduce errors, which could also be a contributing factor to the inconsistent findings observed in previous studies. A few studies reported the associations between alterations of neutrophils, lymphocytes, NLR, PLR, granulocyte-to-lymphocyte ratio (GLR), LMR, and SII with risk of dementia incidence [10, 59, 60]. Our results further corroborate previous findings. Remarkably, Our results also indicated pronounced sexual dimorphism. Importantly, neutrophils exhibit a statistically significant association with dementia in males (HR 1.10, *p* < 0.001) but not in females (HR 1.05, *p* = 0.249). Similarly, the NLR is significantly associated with dementia in males (HR 1.03, *p* = 0.001) but not in females (HR 1.01, *p* = 0.856). These observations suggest that immune system markers may play different roles in the pathogenesis of brain disorders between the sexes. Potential explanations may involve the influence of sex hormones on immune function. Estrogen is known to generally enhance immune responses, while testosterone exerts immunosuppressive effects [72]. These findings underscore the need to consider sex-specific responses in clinical risk assessment and potentially in the tailoring of therapeutic interventions. Regarding PD, three prospective studies examined the association between peripheral immunity markers and incident PD [16, 18, 73], our results showed consistency with two studies that reduced lymphocyte count was associated with an elevated risk of developing PD [16, 73]. Moreover, our research further extended the positive associations to platelet, NLR and LMR.

As for stroke, previous cross-sectional studies have investigated the association between peripheral immunity and stroke [74–76], but the prospective effect on stroke incidence has rarely been addressed. Only two studies explored the relationship between CRP and risk of stroke [23, 77]. Our results confirmed previous findings, and for the first time, provided evidence of associations between neutrophils and monocytes with risk of stroke incidence, which may offer new insights in the etiology and therapy of stroke.

As for psychiatric brain disorders, many cross-sectional studies showed an increased inflammatory response in individuals with MDD, anxiety, BPAD and schizophrenia [78–81]. Additionally, elevated CRP has been found to be correlated with increased depressive symptoms [81], symptom severity [82] and poorer treatment response in individuals with MDD [83, 84]. However, limited studies have reported associations between peripheral immunity markers and MDD incidence [32, 85]. Our study utilized more rigorous inclusion and exclusion criteria, such as excluding individuals with diseases that could potentially affect peripheral immune cells and excluding individuals with a follow-up period of less than 5 years, providing further confirmation of previous findings. Notably, this association was observed specifically in younger individuals, which may arise from variations in the inflammatory processes implicated in depression between younger adults and older adults [86, 87]. We did not observe any significant correlation between inflammatory factors and the risk of schizophrenia and bipolar disorder. Additional research is necessary to confirm these findings.

Since inflammation plays a prominent role in the development of different neuropsychiatric diseases, anti-inflammatory drugs are likely to improve symptoms. Evidence from recent meta-analyses suggests that adjunctive anti-inflammatory medication might be effective in the treatment of mood disorders [88, 89]. However, the results are inconsistent [90, 91]. Our findings provide evidence that early intervention on the systemic inflammation may be a promising therapy to reduce the risk of neuropsychiatric diseases.

Our study possesses several significant strengths. Firstly, we employed a longitudinal rather than cross-sectional approach to examine the relationships between nine peripheral immunity markers and the risk of seven prevalent neurological and psychiatric brain disorders. Secondly, brain imaging data in UKB offers us a chance to investigate the underlying mechanisms influenced by brain microstructural and macrostructural changes. Thirdly, our study benefits from substantial sample size and a prolonged follow-up duration, enhancing the statistical power and enabling us to capture long-term trends and outcomes. We also conducted a comprehensive assessment of various covariates, which allows for the control of potential confounding factors and strengthens the validity of our findings. Fourthly, our analysis using the RCS model identified crucial non-linear associations between immune markers and brain disorders that linear models did not capture. For instance, while the Cox regression analysis identified that higher neutrophil counts were linearly associated with an increased risk of incident dementia, stroke, and MDD, RCS allowed for the detection of non-linear trends in these associations (*p* for non-linearity = 0.002, 0.007, and 0.014, respectively). The U-shaped relationship observed between neutrophil counts and stroke or dementia indicates a potential optimal neutrophil count range, and deviations on either side could increase the risk for these disorders. Clinically, this could lead to the development of targeted therapies that aim to maintain neutrophil counts within a healthy range. Lastly, our findings gain credibility through the utilization of precise and validated diagnoses derived from hospital inpatient or primary care records. Our study has several limitations. Firstly, the sample of UKB was restricted to middle-aged and European ancestry participants, therefore the generalizability of our findings to broader populations may be limited. Secondly, potential confounding may still impact our findings even though we have performed extensive covariate correction. Thirdly, as an observational study, we cannot establish a causal relationship between peripheral immunity and the various brain disorders examined. Lastly, the assessment of peripheral immunity markers and confounding variables was only conducted at baseline, and we lack information on potential changes of these factors over time, which could have influenced our findings.

## Conclusion

To date, our study stands out as the largest longitudinal study that comprehensively explores the association between peripheral immunity markers and incident brain disorders. The consistent findings of elevated innate immunity with increased risk of neurological and psychiatric disorders suggest that abnormal innate immunity is not specific to any particular brain disorder but may reflect a pathological process that leads to brain dysfunction. These findings support the hypothesis that neuroinflammation is important to the etiology of neurological and psychiatric disorders. More research is needed to further elucidate the specific neuroinflammatory mechanisms that give rise to specific disorders.

## Availability of data and materials

The dataset supporting the conclusions of this article is available in the UK Biobank (https://biobank.ndph.ox.ac.uk/showcase/index.cgi) upon application.

### Supplementary information

Additional file 1
