# Supplementary material for: Peripheral immunity and risk of incident brain disorders: a prospective cohort study of 161,968 participants
Source: Transl Psychiatry. 2023 Dec 9;13:382. doi: 10.1038/s41398-023-02683-0 (PMC10710500; doi:10.1038/s41398-023-02683-0)
Supplement: Supplementary file 1 — Additional file 1 [file 41398_2023_2683_MOESM1_ESM.docx]

**Additional file 1**

**Table S1.** Peripheral immunity markers and adjusted covariates with corresponding field ID in UK Biobank. **2**

**Table S2.** Covariates multicollinearity test results**. 3**

**Table S3.** Adjusted HRs for brain disorder incidents in two sensitivity analyses. **5**

**Table S4.** Adjusted HRs for brain disorder incidents stratified by age at baseline. **7**

**Table S5.** Adjusted HRs for brain disorder incidents stratified by sex. **9**

**Table S6.** Associations between peripheral immunity markers and FA (fractional anisotropy) values of white matter tracts **11**

**Table S7.** Associations between peripheral immunity markers and MD (mean diffusivity) values of white matter tracts **13**

**Table S8.** Associations between peripheral immunity markers and the volume of cortical and subcortical regions **16**

**Table S9.** Associations between peripheral immunity markers and the area of cortical regions **18**

**Table S10.**  Associations between peripheral immunity markers and the thickness of cortical regions **19**

**Table S1.** Peripheral immunity markers and adjusted covariates with corresponding field ID in UK Biobank

| **Markers** | **Field ID** |
| --- | --- |
| Neutrophil count | 30140 |
| Lymphocyte count | 30120 |
| Monocyte count | 30130 |
| Platelet count | 30080 |
| C-reactive protein | 30710 |
| Age | 21022 |
| Sex | 31 |
| Ethnicity | 21000 |
| Education | 6138 |
| Systolic blood pressure | 93; 4080 |
| Diastolic blood pressure | 94; 4079 |

**Table S2.** Covariates multicollinearity test results

| VIF | Age | Sex | Ethnicity | Education | SBP | DBP |
| --- | --- | --- | --- | --- | --- | --- |
| **Dementia** |  |  |  |  |  |  |
| Lymphocyte | 1.0969 | 1.0313 | 1.0040 | 1.0142 | 1.9840 | 1.9522 |
| Monocyte | 1.0997 | 1.0707 | 1.0031 | 1.0144 | 1.9842 | 1.9522 |
| Neutrophil | 1.0987 | 1.0322 | 1.0043 | 1.0189 | 1.9894 | 1.9522 |
| Platelet | 1.0998 | 1.0964 | 1.0026 | 1.0143 | 1.9845 | 1.9527 |
| CRP | 1.0980 | 1.0281 | 1.0022 | 1.0164 | 1.9839 | 1.9542 |
| NLR | 1.0993 | 1.0444 | 1.0057 | 1.0141 | 1.9859 | 1.9531 |
| LMR | 1.0976 | 1.0476 | 1.0044 | 1.0136 | 1.9839 | 1.9521 |
| PLR | 1.0968 | 1.0272 | 1.0036 | 1.0137 | 1.9840 | 1.9522 |
| SII | 1.0974 | 1.0269 | 1.0051 | 1.0144 | 1.9862 | 1.9525 |
| **PD** |  |  |  |  |  |  |
| Lymphocyte | 1.0969 | 1.0313 | 1.0040 | 1.0142 | 1.9840 | 1.9522 |
| Monocyte | 1.0997 | 1.0707 | 1.0031 | 1.0144 | 1.9842 | 1.9522 |
| Neutrophil | 1.0987 | 1.0322 | 1.0043 | 1.0189 | 1.9894 | 1.9522 |
| Platelet | 1.0998 | 1.0964 | 1.0026 | 1.0143 | 1.9845 | 1.9527 |
| CRP | 1.0980 | 1.0281 | 1.0022 | 1.0164 | 1.9839 | 1.9542 |
| NLR | 1.0993 | 1.0444 | 1.0057 | 1.0141 | 1.9859 | 1.9531 |
| LMR | 1.0976 | 1.0476 | 1.0044 | 1.0136 | 1.9839 | 1.9521 |
| PLR | 1.0968 | 1.0272 | 1.0036 | 1.0137 | 1.9840 | 1.9522 |
| SII | 1.0974 | 1.0269 | 1.0051 | 1.0144 | 1.9862 | 1.9525 |
| **Stroke** |  |  |  |  |  |  |
| Lymphocyte | 1.0969 | 1.0313 | 1.0040 | 1.0142 | 1.9840 | 1.9522 |
| Monocyte | 1.0997 | 1.0707 | 1.0031 | 1.0144 | 1.9842 | 1.9522 |
| Neutrophil | 1.0987 | 1.0322 | 1.0043 | 1.0189 | 1.9894 | 1.9522 |
| Platelet | 1.0998 | 1.0964 | 1.0026 | 1.0143 | 1.9845 | 1.9527 |
| CRP | 1.0980 | 1.0281 | 1.0022 | 1.0164 | 1.9839 | 1.9542 |
| NLR | 1.0993 | 1.0444 | 1.0057 | 1.0141 | 1.9859 | 1.9531 |
| LMR | 1.0976 | 1.0476 | 1.0044 | 1.0136 | 1.9839 | 1.9521 |
| PLR | 1.0968 | 1.0272 | 1.0036 | 1.0137 | 1.9840 | 1.9522 |
| SII | 1.0974 | 1.0269 | 1.0051 | 1.0144 | 1.9862 | 1.9525 |
| **MDD** |  |  |  |  |  |  |
| Lymphocyte | 1.0969 | 1.0313 | 1.0040 | 1.0142 | 1.9840 | 1.9522 |
| Monocyte | 1.0997 | 1.0707 | 1.0031 | 1.0144 | 1.9842 | 1.9522 |
| Neutrophil | 1.0987 | 1.0322 | 1.0043 | 1.0189 | 1.9894 | 1.9522 |
| Platelet | 1.0998 | 1.0964 | 1.0026 | 1.0143 | 1.9845 | 1.9527 |
| CRP | 1.0980 | 1.0281 | 1.0022 | 1.0164 | 1.9839 | 1.9542 |
| NLR | 1.0993 | 1.0444 | 1.0057 | 1.0141 | 1.9859 | 1.9531 |
| LMR | 1.0976 | 1.0476 | 1.0044 | 1.0136 | 1.9839 | 1.9521 |
| PLR | 1.0968 | 1.0272 | 1.0036 | 1.0137 | 1.9840 | 1.9522 |
| SII | 1.0974 | 1.0269 | 1.0051 | 1.0144 | 1.9862 | 1.9525 |
| **Anxiety** |  |  |  |  |  |  |
| Lymphocyte | 1.0969 | 1.0313 | 1.0040 | 1.0142 | 1.9840 | 1.9522 |
| Monocyte | 1.0997 | 1.0707 | 1.0031 | 1.0144 | 1.9842 | 1.9522 |
| Neutrophil | 1.0987 | 1.0322 | 1.0043 | 1.0189 | 1.9894 | 1.9522 |
| Platelet | 1.0998 | 1.0964 | 1.0026 | 1.0143 | 1.9845 | 1.9527 |
| CRP | 1.0980 | 1.0281 | 1.0022 | 1.0164 | 1.9839 | 1.9542 |
| NLR | 1.0993 | 1.0444 | 1.0057 | 1.0141 | 1.9859 | 1.9531 |
| LMR | 1.0976 | 1.0476 | 1.0044 | 1.0136 | 1.9839 | 1.9521 |
| PLR | 1.0968 | 1.0272 | 1.0036 | 1.0137 | 1.9840 | 1.9522 |
| SII | 1.0974 | 1.0269 | 1.0051 | 1.0144 | 1.9862 | 1.9525 |
| **BPAD** |  |  |  |  |  |  |
| Lymphocyte | 1.0969 | 1.0313 | 1.0040 | 1.0142 | 1.9840 | 1.9522 |
| Monocyte | 1.0997 | 1.0707 | 1.0031 | 1.0144 | 1.9842 | 1.9522 |
| Neutrophil | 1.0987 | 1.0322 | 1.0043 | 1.0189 | 1.9894 | 1.9522 |
| Platelet | 1.0998 | 1.0964 | 1.0026 | 1.0143 | 1.9845 | 1.9527 |
| CRP | 1.0980 | 1.0281 | 1.0022 | 1.0164 | 1.9839 | 1.9542 |
| NLR | 1.0993 | 1.0444 | 1.0057 | 1.0141 | 1.9859 | 1.9531 |
| LMR | 1.0976 | 1.0476 | 1.0044 | 1.0136 | 1.9839 | 1.9521 |
| PLR | 1.0968 | 1.0272 | 1.0036 | 1.0137 | 1.9840 | 1.9522 |
| SII | 1.0974 | 1.0269 | 1.0051 | 1.0144 | 1.9862 | 1.9525 |
| **Schizophrenia** |  |  |  |  |  |  |
| Lymphocyte | 1.0969 | 1.0313 | 1.0040 | 1.0142 | 1.9840 | 1.9522 |
| Monocyte | 1.0997 | 1.0707 | 1.0031 | 1.0144 | 1.9842 | 1.9522 |
| Neutrophil | 1.0987 | 1.0322 | 1.0043 | 1.0189 | 1.9894 | 1.9522 |
| Platelet | 1.0998 | 1.0964 | 1.0026 | 1.0143 | 1.9845 | 1.9527 |
| CRP | 1.0980 | 1.0281 | 1.0022 | 1.0164 | 1.9839 | 1.9542 |
| NLR | 1.0993 | 1.0444 | 1.0057 | 1.0141 | 1.9859 | 1.9531 |
| LMR | 1.0976 | 1.0476 | 1.0044 | 1.0136 | 1.9839 | 1.9521 |
| PLR | 1.0968 | 1.0272 | 1.0036 | 1.0137 | 1.9840 | 1.9522 |
| SII | 1.0974 | 1.0269 | 1.0051 | 1.0144 | 1.9862 | 1.9525 |

Abbreviations: VIF, variance inflation factor; SBP, systolic blood pressure; DBP, diastolic blood pressure; CRP, C-reactive protein; NLR, neutrophils/lymphocytes ratio; LMR, lymphocytes/monocytes ratio; PLR, platelets/lymphocytes ratio; SII, systemic immune-inflammation index (neutrophils×platelets/lymphocytes); PD, Parkinson’s disease; MDD, major depressive disorder; BPAD, bipolar affective disorder.

#

**Table S3.** Adjusted HRs for brain disorder incidents in two sensitivity analyses

|  | **Extreme values excluded** | | **Imputing missing values** | |
| --- | --- | --- | --- | --- |
| **Dementia** |  |  |  |  |
| Lymphocyte | 1.00 (0.97-1.03) | 0.886 | 0.99 (0.96-1.03) | 0.803 |
| Monocyte | 1.00 (0.96-1.03) | 0.886 | 1.00 (0.96-1.03) | 0.826 |
| Neutrophil | 1.08 (1.04-1.12) | **<0.001** | 1.08 (1.05-1.12) | **<0.001** |
| Platelet | 0.97 (0.93-1.01) | 0.236 | 0.98 (0.95-1.02) | 0.526 |
| CRP | 0.94 (0.91-0.98) | **0.019** | 0.95 (0.91-0.99) | **0.015** |
| NLR | 1.07 (1.03-1.11) | **<0.001** | 1.03 (1.02-1.05) | **<0.001** |
| LMR | 0.99 (0.95-1.03) | 0.886 | 0.99 (0.95-1.03) | 0.803 |
| PLR | 0.99 (0.94-1.04) | 0.886 | 1.00 (0.98-1.03) | 0.803 |
| SII | 1.04 (1.01-1.08) | **0.029** | 1.03 (1.01-1.05) | **0.004** |
| **PD** |  |  |  |  |
| Lymphocyte | 0.73 (0.66-0.81) | **<0.001** | 0.74 (0.67-0.82) | **<0.001** |
| Monocyte | 0.90 (0.84-0.97) | **0.008** | 0.92 (0.86-0.98) | **0.016** |
| Neutrophil | 1.03 (0.97-1.09) | 0.357 | 1.02 (0.97-1.08) | 0.478 |
| Platelet | 0.88 (0.83-0.94) | **<0.001** | 0.88 (0.83-0.94) | **<0.001** |
| CRP | 0.93 (0.87-1.00) | **0.047** | 0.93 (0.87-0.99) | **0.029** |
| NLR | 1.17 (1.11-1.23) | **<0.001** | 1.04 (1.03-1.06) | **<0.001** |
| LMR | 0.80 (0.68-0.95) | **0.015** | 0.79 (0.67-0.94) | **0.013** |
| PLR | 1.01 (1.00-1.03) | 0.150 | 1.01 (1.00-1.03) | 0.083 |
| SII | 1.08 (1.02-1.14) | **0.008** | 1.04 (1.01-1.06) | **0.013** |
| **Stroke** |  |  |  |  |
| Lymphocyte | 1.01 (1.00-1.03) | 0.256 | 1.01 (0.99-1.03) | 0.279 |
| Monocyte | 1.05 (1.03-1.07) | **<0.001** | 1.05 (1.03-1.07) | **<0.001** |
| Neutrophil | 1.06 (1.03-1.09) | **<0.001** | 1.06 (1.03-1.08) | **<0.001** |
| Platelet | 1.02 (0.99-1.05) | 0.278 | 1.02 (0.99-1.05) | 0.273 |
| CRP | 1.04 (1.02-1.07) | **0.002** | 1.04 (1.01-1.06) | **0.010** |
| NLR | 1.01 (0.98-1.04) | 0.404 | 1.01 (0.99-1.03) | 0.375 |
| LMR | 0.98 (0.94-1.02) | 0.368 | 0.97 (0.93-1.01) | 0.273 |
| PLR | 0.97 (0.93-1.01) | 0.256 | 0.98 (0.95-1.02) | 0.375 |
| SII | 1.02 (0.99-1.05 | 0.260 | 1.02 (0.99-1.04) | 0.273 |
| **MDD** |  |  |  |  |
| Lymphocyte | 1.03 (1.01-1.04) | **<0.001** | 1.03 (1.01-1.04) | **<0.001** |
| Monocyte | 1.04 (1.02-1.07) | **<0.001** | 1.04 (1.02-1.07) | **<0.001** |
| Neutrophil | 1.13 (1.10-1.17) | **<0.001** | 1.13 (1.10-1.16) | **<0.001** |
| Platelet | 1.06 (1.03-1.1) | **<0.001** | 1.06 (1.03-1.09) | **<0.001** |
| CRP | 1.05 (1.02-1.08) | **<0.001** | 1.06(1.03-1.09) | **<0.001** |
| NLR | 1.03 (1.00-1.07) | 0.072 | 1.03 (1.01-1.05) | **0.001** |
| LMR | 0.99 (0.95-1.03) | 0.580 | 1.00 (0.97-1.03) | 0.941 |
| PLR | 0.93 (0.89-0.98) | **0.007** | 0.99 (0.95-1.03) | 0.595 |
| SII | 1.05 (1.02-1.08) | **0.003** | 1.04 (1.02-1.06) | **<0.001** |
| **Anxiety** |  |  |  |  |
| Lymphocyte | 1.00 (0.98-1.03) | 0.794 | 1.01 (0.98-1.03) | 0.879 |
| Monocyte | 1.02 (1.00-1.05) | 0.215 | 1.02 (1.00-1.05) | 0.198 |
| Neutrophil | 1.07 (1.04-1.10) | **<0.001** | 1.07 (1.05-1.10) | **<0.001** |
| Platelet | 1.00 (0.97-1.03) | 0.870 | 1.00 (0.97-1.03) | 0.879 |
| CRP | 0.99 (0.97-1.02) | 0.794 | 1.00 (0.97-1.02) | 0.879 |
| NLR | 1.03 (1.00-1.06) | 0.159 | 1.03 (1.01-1.05) | **0.008** |
| LMR | 0.98 (0.94-1.02) | 0.432 | 1.00 (0.98-1.03) | 0.879 |
| PLR | 0.97 (0.93-1.01) | 0.215 | 0.99 (0.96-1.03) | 0.879 |
| SII | 1.02 (1.00-1.05) | 0.215 | 1.03 (1.01-1.05) | **0.009** |
| **BPAD** |  |  |  |  |
| Lymphocyte | 0.96 (0.67-1.37) | 0.816 | 0.79 (0.52-1.21) | 0.564 |
| Monocyte | 0.92 (0.67-1.26) | 0.771 | 0.90 (0.67-1.22) | 0.564 |
| Neutrophil | 1.22 (0.97-1.53) | 0.742 | 1.15 (0.93-1.41) | 0.564 |
| Platelet | 0.86 (0.66-1.12) | 0.771 | 0.89 (0.69-1.14) | 0.564 |
| CRP | 0.91 (0.67-1.25) | 0.771 | 0.90 (0.66-1.22) | 0.564 |
| NLR | 1.18 (0.94-1.48) | 0.742 | 1.05 (0.98-1.12) | 0.564 |
| LMR | 0.90 (0.49-1.65) | 0.816 | 0.75 (0.36-1.56) | 0.564 |
| PLR | 0.87 (0.60-1.27) | 0.771 | 1.01 (0.94-1.09) | 0.735 |
| SII | 1.08 (0.85-1.37) | 0.771 | 1.05 (0.95-1.15) | 0.564 |
| **Schizophrenia** |  |  |  |  |
| Lymphocyte | 1.02 (0.85-1.22) | 0.842 | 0.99 (0.73-1.33) | 0.927 |
| Monocyte | 1.07 (0.93-1.24) | 0.585 | 1.06 (0.88-1.26) | 0.624 |
| Neutrophil | 1.22 (0.94-1.58) | 0.585 | 1.24 (1.10-1.41) | **0.006** |
| Platelet | 1.23 (0.93-1.62) | 0.585 | 1.29 (1.04-1.6) | 0.081 |
| CRP | 1.07 (0.86-1.34) | 0.731 | 1.09 (0.90-1.32) | 0.558 |
| NLR | 1.08 (0.83-1.41) | 0.731 | 1.04 (0.98-1.1) | 0.423 |
| LMR | 0.60 (0.23-1.58) | 0.585 | 0.57 (0.22-1.45) | 0.423 |
| PLR | 1.01 (0.93-1.09) | 0.842 | 1.02 (0.97-1.07) | 0.624 |
| SII | 1.18 (0.91-1.52) | 0.585 | 1.05 (1.00-1.10) | 0.129 |

Note: Two sensitivity analyses were performed: (1) exclusion of participants with the extreme values (> mean ± 3SD) of all exposures, (2) imputing missing values for all exposures. Analyses were adjusted by age at baseline, sex, ethnicity, education, SBP and DBP. The P values were corrected by false-discovery rate (FDR).

Abbreviations: HR, hazard ratio; CRP, C-reactive protein; NLR, neutrophils/lymphocytes ratio; LMR, lymphocytes/monocytes ratio; PLR, platelets/lymphocytes ratio; SII, systemic immune-inflammation index (neutrophils×platelets/lymphocytes); PD, Parkinson’s disease; MDD, major depressive disorder; BPAD, bipolar affective disorder.

**Table S4.** Adjusted HRs for brain disorder incidents stratified by age at baseline

|  | **Male** | | **Female** | |
| --- | --- | --- | --- | --- |
| **Peripheral immunity** | **HR (95%CI)** | ***p-value*** | **HR (95%CI)** | ***p-value*** |
| **Dementia** |  |  |  |  |
| Lymphocyte | 0.99 (0.94-1.04) | 0.865 | 1.01 (0.98-1.05) | 0.856 |
| Monocyte | 1.00 (0.95-1.05) | 0.947 | 1.00 (0.95-1.05) | 0.930 |
| Neutrophil | 1.10 (1.05-1.15) | **<0.001** | 1.05 (1.00-1.11) | 0.249 |
| Platelet | 1.00 (0.95-1.05) | 0.947 | 0.96 (0.91-1.01 | 0.293 |
| CRP | 0.97 (0.92-1.02) | 0.391 | 0.93 (0.87-0.99) | 0.140 |
| NLR | 1.03 (1.02-1.05) | **0.001** | 1.01 (0.97-1.06) | 0.856 |
| LMR | 0.99 (0.93-1.05) | 0.865 | 0.99 (0.93-1.05) | 0.930 |
| PLR | 1.02 (0.99-1.05) | 0.391 | 0.94 (0.86-1.03) | 0.470 |
| SII | 1.04 (1.02-1.06) | **0.002** | 1.00 (0.95-1.05) | 0.930 |
| **PD** |  |  |  |  |
| Lymphocyte | 0.68 (0.59-0.79) | **<0.001** | 0.81 (0.69-0.94) | **0.031** |
| Monocyte | 0.88 (0.81-0.96) | **0.012** | 0.97 (0.88-1.08) | 0.717 |
| Neutrophil | 1.03 (0.96-1.11) | 0.356 | 1.00 (0.91-1.10) | 0.994 |
| Platelet | 0.89 (0.83-0.96) | **0.007** | 0.89 (0.81-0.99) | 0.090 |
| CRP | 0.94 (0.86-1.02) | 0.147 | 0.92 (0.81-1.03) | 0.225 |
| NLR | 1.04 (1.02-1.06) | **<0.001** | 1.05 (1.02-1.09) | **0.031** |
| LMR | 0.82 (0.69-0.97) | **0.040** | 0.78 (0.56-1.08) | 0.225 |
| PLR | 1.03 (1.00-1.06) | 0.064 | 1.01 (0.99-1.04) | 0.484 |
| SII | 1.03 (1.00-1.07) | 0.064 | 1.04 (0.99-1.10) | 0.182 |
| **Stroke** |  |  |  |  |
| Lymphocyte | 1.01 (0.99-1.04) | 0.454 | 1.01 (0.99-1.04) | 0.495 |
| Monocyte | 1.05 (1.02-1.08) | **0.012** | 1.05 (1.03-1.07) | **<0.001** |
| Neutrophil | 1.05 (1.02-1.09) | **0.017** | 1.07 (1.02-1.11) | **0.010** |
| Platelet | 1.00 (0.97-1.04) | 0.838 | 1.04 (0.99-1.08) | 0.233 |
| CRP | 1.04 (1.01-1.08) | **0.018** | 1.03 (0.99-1.07) | 0.233 |
| NLR | 1.01 (0.98-1.04) | 0.616 | 1.01 (0.97-1.05) | 0.778 |
| LMR | 0.96 (0.91-1.02) | 0.454 | 0.98 (0.93-1.04) | 0.656 |
| PLR | 0.98 (0.94-1.02) | 0.454 | 0.97 (0.91-1.05) | 0.605 |
| SII | 1.01 (0.98-1.04) | 0.616 | 1.02 (0.98-1.05) | 0.495 |
| **MDD** |  |  |  |  |
| Lymphocyte | 1.03 (1.01-1.05) | **0.003** | 1.02 (1.00-1.04) | **0.084** |
| Monocyte | 1.05 (1.01-1.09) | **0.031** | 1.04 (1.01-1.07) | **0.004** |
| Neutrophil | 1.17 (1.12-1.21) | **<0.001** | 1.09 (1.05-1.13) | **<0.001** |
| Platelet | 1.09 (1.04-1.14) | **0.001** | 1.05 (1.01-1.10) | **0.029** |
| CRP | 1.05 (1.01-1.09) | **0.032** | 1.06 (1.03-1.10) | **0.003** |
| NLR | 1.03 (1.00-1.05) | **0.031** | 1.03 (1.00-1.06) | 0.065 |
| LMR | 1.01 (0.98-1.05) | 0.424 | 0.96 (0.89-1.03) | 0.275 |
| PLR | 0.98 (0.92-1.03) | 0.424 | 1.00 (0.95-1.04) | 0.930 |
| SII | 1.04 (1.02-1.06) | **0.002** | 1.04 (1.02-1.07) | **0.004** |
| **Anxiety** |  |  |  |  |
| Lymphocyte | 1.00 (0.97-1.05) | 0.913 | 1.00 (0.97-1.03) | 0.997 |
| Monocyte | 1.01 (0.97-1.06) | 0.847 | 1.03 (1.00-1.05) | 0.126 |
| Neutrophil | 1.08 (1.03-1.13) | **0.017** | 1.06 (1.03-1.10) | **0.003** |
| Platelet | 0.98 (0.93-1.03) | 0.720 | 1.01 (0.98-1.05) | 0.855 |
| CRP | 0.98 (0.93-1.03) | 0.720 | 1.00 (0.97-1.04) | 0.997 |
| NLR | 1.02 (0.98-1.05) | 0.720 | 1.03 (1.01-1.06) | **0.020** |
| LMR | 1.00 (0.95-1.05) | 0.913 | 0.97 (0.91-1.02) | 0.440 |
| PLR | 0.97 (0.92-1.03) | 0.720 | 1.00 (0.97-1.03) | 0.997 |
| SII | 1.01 (0.97-1.05) | 0.894 | 1.04 (1.01-1.07) | **0.013** |
| **BPAD** |  |  |  |  |
| Lymphocyte | 1.03 (0.88-1.21) | 0.902 | 0.71 (0.42-1.20) | 0.774 |
| Monocyte | 0.98 (0.65-1.49) | 0.949 | 0.89 (0.60-1.33) | 0.774 |
| Neutrophil | 1.19 (0.87-1.63) | 0.902 | 1.14 (0.85-1.52) | 0.774 |
| Platelet | 1.10 (0.77-1.58) | 0.902 | 0.76 (0.54-1.07) | 0.774 |
| CRP | 0.80 (0.41-1.54) | 0.902 | 0.94 (0.64-1.36) | 0.820 |
| NLR | 1.04 (0.90-1.19) | 0.902 | 1.05 (0.93-1.19) | 0.774 |
| LMR | 1.01 (0.74-1.38) | 0.949 | 0.64 (0.20-2.08) | 0.774 |
| PLR | 1.03 (0.90-1.19) | 0.902 | 0.85 (0.46-1.57) | 0.774 |
| SII | 1.05 (0.92-1.19) | 0.902 | 1.00 (0.74-1.35) | 0.986 |
| **Schizophrenia** |  |  |  |  |
| Lymphocyte | 0.93 (0.52-1.66) | 0.904 | 1.02 (0.76-1.36) | 0.918 |
| Monocyte | 0.80 (0.50-1.27) | 0.718 | 1.09 (1.00-1.18) | 0.098 |
| Neutrophil | 1.20 (0.94-1.53) | 0.718 | 1.47 (1.15-1.87) | **0.020** |
| Platelet | 1.17 (0.86-1.60) | 0.718 | 1.42 (1.03-1.96) | 0.098 |
| CRP | 0.99 (0.69-1.42) | 0.944 | 1.14 (0.88-1.48) | 0.423 |
| NLR | 1.03 (0.95-1.12) | 0.718 | 1.07 (0.97-1.18) | 0.251 |
| LMR | 0.86 (0.38-1.93) | 0.904 | 0.15 (0.02-1.21) | 0.135 |
| PLR | 1.03 (0.95-1.11) | 0.718 | 1.01 (0.93-1.11) | 0.847 |
| SII | 1.04 (0.97-1.12) | 0.718 | 1.09 (1.00-1.17) | 0.098 |

Note: Analyses were adjusted by age at baseline, sex, ethnicity, education, SBP and DBP. The P values were corrected by false-discovery rate (FDR).

Abbreviations: HR, hazard ratio; CRP, C-reactive protein; NLR, neutrophils/lymphocytes ratio; LMR, lymphocytes/monocytes ratio; PLR, platelets/lymphocytes ratio; SII, systemic immune-inflammation index (neutrophils×platelets/lymphocytes); PD, Parkinson’s disease; MDD, major depressive disorder; BPAD, bipolar affective disorder.

**Table S5.** Adjusted HRs for brain disorder incidents stratified by sex

|  | **55–65 years** | | **≥ 65 years** | |
| --- | --- | --- | --- | --- |
| **Peripheral immunity** | **HR (95%CI)** | ***p-value*** | **HR (95%CI)** | ***p-value*** |
| **Dementia** |  |  |  |  |
| Lymphocyte | 1.01 (0.97-1.04) | 0.872 | 0.99 (0.93-1.04) | 0.671 |
| Monocyte | 1.00 (0.95-1.06) | 0.903 | 0.99 (0.94-1.05) | 0.721 |
| Neutrophil | 1.08 (1.03-1.14) | **0.012** | 1.08 (1.03-1.13) | **0.013** |
| Platelet | 0.97 (0.92-1.03) | 0.560 | 0.98 (0.93-1.04) | 0.671 |
| CRP | 0.95 (0.89-1.01) | 0.340 | 0.95 (0.89-1.00) | 0.148 |
| NLR | 1.03 (0.99-1.07) | 0.474 | 1.04 (1.01-1.06) | **0.010** |
| LMR | 1.00 (0.96-1.05) | 0.903 | 0.95 (0.87-1.04) | 0.440 |
| PLR | 0.96 (0.91-1.02) | 0.487 | 1.01 (0.98-1.04) | 0.671 |
| SII | 1.01 (0.97-1.06) | 0.817 | 1.04 (1.01-1.06) | **0.019** |
| **PD** |  |  |  |  |
| Lymphocyte | 0.70 (0.60-0.80) | **<0.001** | 0.79 (0.68-0.92) | **0.025** |
| Monocyte | 0.85 (0.78-0.94) | **0.003** | 0.98 (0.90-1.08) | 0.746 |
| Neutrophil | 0.99 (0.92-1.07) | 0.802 | 1.07 (0.98-1.16) | 0.248 |
| Platelet | 0.89 (0.82-0.97) | **0.011** | 0.89 (0.80-0.97) | **0.039** |
| CRP | 0.92 (0.84-1.01) | 0.123 | 0.94 (0.84-1.04) | 0.317 |
| NLR | 1.06 (1.04-1.09) | **<0.001** | 1.04 (1.01-1.07) | **0.039** |
| LMR | 0.83 (0.66-1.05) | 0.128 | 0.75 (0.59-0.96) | **0.046** |
| PLR | 1.05 (1.02-1.08) | **0.003** | 1.01 (0.96-1.06) | 0.744 |
| SII | 1.05 (1.01-1.09) | **0.012** | 1.03 (0.98-1.08) | 0.317 |
| **Stroke** |  |  |  |  |
| Lymphocyte | 1.02 (1.00-1.04) | 0.069 | 0.99 (0.94-1.04) | 0.638 |
| Monocyte | 1.05 (1.03-1.07) | **<0.001** | 1.05 (1.02-1.09) | **0.013** |
| Neutrophil | 1.06 (1.02-1.10) | **0.005** | 1.06 (1.01-1.11) | 0.052 |
| Platelet | 1.02 (0.98-1.05) | 0.558 | 1.02 (0.98-1.07) | 0.458 |
| CRP | 1.05 (1.02-1.08) | **0.010** | 1.03 (0.99-1.07) | 0.366 |
| NLR | 1.00 (0.97-1.04) | 0.994 | 1.02 (0.98-1.05) | 0.458 |
| LMR | 0.99 (0.95-1.03) | 0.817 | 0.91 (0.83-1.01) | 0.197 |
| PLR | 0.96 (0.92-1.00) | 0.081 | 1.00 (0.96-1.05) | 0.975 |
| SII | 1.01 (0.97-1.04) | 0.817 | 1.02 (0.99-1.06) | 0.366 |
| **MDD** |  |  |  |  |
| Lymphocyte | 1.031.01-1.04 | **0.001** | 1.03 (0.99-1.07) | 0.561 |
| Monocyte | 1.051.02-1.08 | **<0.001** | 1.04 (1.00-1.08) | 0.356 |
| Neutrophil | 1.151.11-1.19 | **<0.001** | 1.08 (1.02-1.14) | 0.123 |
| Platelet | 1.091.05-1.13 | **<0.001** | 1.02 (0.96-1.08) | 0.853 |
| CRP | 1.071.04-1.1 | **<0.001** | 1.02 (0.96-1.08) | 0.853 |
| NLR | 1.041.02-1.06 | **<0.001** | 1.01 (0.95-1.07) | 0.880 |
| LMR | 0.980.93-1.03 | 0.467 | 1.00 (0.94-1.06) | 0.981 |
| PLR | 0.990.96-1.03 | 0.759 | 0.97 (0.86-1.10) | 0.853 |
| SII | 1.061.04-1.08 | **<0.001** | 1.02 (0.97-1.07) | 0.853 |
| **Anxiety** |  |  |  |  |
| Lymphocyte | 1.00 (0.97-1.03) | 0.997 | 1.02 (0.97-1.06) | 0.695 |
| Monocyte | 1.02 (0.98-1.05) | 0.641 | 1.04 (1.00-1.08) | 0.197 |
| Neutrophil | 1.06 (1.03-1.10) | **0.002** | 1.09 (1.03-1.14) | **0.017** |
| Platelet | 1.00 (0.96-1.03) | 0.948 | 1.01 (0.96-1.07) | 0.723 |
| CRP | 0.99 (0.96-1.03) | 0.948 | 0.99 (0.94-1.05) | 0.723 |
| NLR | 1.03 (1.01-1.06) | **0.019** | 1.01 (0.97-1.06) | 0.695 |
| LMR | 0.98 (0.94-1.02) | 0.641 | 0.97 (0.90-1.05) | 0.695 |
| PLR | 1.00 (0.97-1.04) | 0.948 | 0.95 (0.84-1.07) | 0.695 |
| SII | 1.04 (1.01-1.06) | **0.019** | 1.02 (0.97-1.06) | 0.695 |
| **BPAD** |  |  |  |  |
| Lymphocyte | 1.00 (0.76-1.31) | 0.973 | 0.71 (0.31-1.63) | 0.996 |
| Monocyte | 1.04 (0.83-1.29) | 0.834 | 0.58 (0.29-1.17) | 0.996 |
| Neutrophil | 1.25 (0.98-1.60) | 0.686 | 0.93 (0.56-1.54) | 0.996 |
| Platelet | 0.88 (0.64-1.20) | 0.834 | 0.90 (0.55-1.48) | 0.996 |
| CRP | 0.83 (0.53-1.30) | 0.834 | 0.99 (0.61-1.61) | 0.996 |
| NLR | 1.06 (0.94-1.18) | 0.834 | 1.04 (0.83-1.29) | 0.996 |
| LMR | 0.76 (0.03-1.92) | 0.834 | 0.98 (0.54-1.79) | 0.996 |
| PLR | 0.95 (0.68-1.32) | 0.834 | 1.01 (0.81-1.26) | 0.996 |
| SII | 1.05 (0.88-1.24) | 0.834 | 1.00 (0.60-1.67) | 0.996 |
| **Schizophrenia** |  |  |  |  |
| Lymphocyte | 0.73 (0.37-1.48) | 0.578 | 1.05 (0.86-1.27) | 0.735 |
| Monocyte | 0.80 (0.49-1.31) | 0.578 | 1.12 (1-1.25) | 0.187 |
| Neutrophil | 1.12 (0.8-1.58) | 0.605 | 1.30 (1.15-1.46) | **<0.001** |
| Platelet | 1.35 (1.07-1.71) | 0.116 | 1.15 (0.78-1.70) | 0.605 |
| CRP | 0.99 (0.67-1.45) | 0.955 | 1.15 (0.88-1.51) | 0.562 |
| NLR | 1.07 (0.94-1.21) | 0.578 | 1.04 (0.96-1.12) | 0.572 |
| LMR | 0.66 (0.18-2.47) | 0.605 | 0.37 (0.09-1.45) | 0.463 |
| PLR | 1.07 (0.99-1.16) | 0.282 | 1.01 (0.75-1.34) | 0.970 |
| SII | 1.08 (0.99-1.19) | 0.282 | 1.05 (0.97-1.13) | 0.504 |

Note: Analyses were adjusted by age at baseline, sex, ethnicity, education, SBP and DBP. The P values were corrected by false-discovery rate (FDR).

Abbreviations: HR, hazard ratio; CRP, C-reactive protein; NLR, neutrophils/lymphocytes ratio; LMR, lymphocytes/monocytes ratio; PLR, platelets/lymphocytes ratio; SII, systemic immune-inflammation index (neutrophils×platelets/lymphocytes); PD, Parkinson’s disease; MDD, major depressive disorder; BPAD, bipolar affective disorder; SBP, systolic blood pressure; DBP, diastolic blood pressure.

**Table S6.** Associations between peripheral immunity markers and FA (fractional anisotropy) values of white matter tracts

| **Predictor** | **Outcome** | **ES** | **Adjusted_p** |
| --- | --- | --- | --- |
| CRP | Mean FA in fornix cres+stria terminalis on FA skeleton (left) | -0.009 | 0.038 |
| CRP | Mean FA in genu of corpus callosum on FA skeleton | -0.010 | 0.004 |
| CRP | Mean FA in superior cerebellar peduncle on FA skeleton (right) | -0.009 | 0.039 |
| Neutrophil | Mean FA in anterior corona radiata on FA skeleton (right) | -0.031 | 0.004 |
| Neutrophil | Mean FA in fornix on FA skeleton | -0.026 | 0.049 |
| Neutrophil | Mean FA in genu of corpus callosum on FA skeleton | -0.031 | 0.005 |
| Neutrophil | Mean FA in superior cerebellar peduncle on FA skeleton (right) | -0.029 | 0.015 |
| Neutrophil | Mean FA in superior fronto-occipital fasciculus on FA skeleton (left) | -0.038 | 0.000 |
| Neutrophil | Mean FA in superior fronto-occipital fasciculus on FA skeleton (right) | -0.028 | 0.027 |
| PLR | Mean FA in external capsule on FA skeleton (left) | -0.001 | 0.005 |
| Platelet | Mean FA in anterior corona radiata on FA skeleton (left) | -0.001 | 0.001 |
| Platelet | Mean FA in anterior corona radiata on FA skeleton (right) | -0.001 | 0.000 |
| Platelet | Mean FA in anterior limb of internal capsule on FA skeleton (left) | -0.001 | 0.001 |
| Platelet | Mean FA in anterior limb of internal capsule on FA skeleton (right) | -0.001 | 0.027 |
| Platelet | Mean FA in cerebral peduncle on FA skeleton (left) | -0.001 | 0.001 |
| Platelet | Mean FA in cerebral peduncle on FA skeleton (right) | -0.001 | 0.001 |
| Platelet | Mean FA in genu of corpus callosum on FA skeleton | -0.001 | 0.007 |
| Platelet | Mean FA in medial lemniscus on FA skeleton (right) | -0.001 | 0.013 |
| Platelet | Mean FA in posterior limb of internal capsule on FA skeleton (left) | -0.001 | 0.018 |
| Platelet | Mean FA in superior cerebellar peduncle on FA skeleton (left) | -0.001 | 0.031 |
| Platelet | Mean FA in superior fronto-occipital fasciculus on FA skeleton (left) | -0.001 | 0.001 |
| Platelet | Mean FA in superior fronto-occipital fasciculus on FA skeleton (right) | -0.001 | 0.000 |
| SII | Mean FA in anterior corona radiata on FA skeleton (left) | 0.000 | 0.002 |
| SII | Mean FA in anterior corona radiata on FA skeleton (right) | 0.000 | 0.000 |
| SII | Mean FA in anterior limb of internal capsule on FA skeleton (left) | 0.000 | 0.008 |
| SII | Mean FA in anterior limb of internal capsule on FA skeleton (right) | 0.000 | 0.029 |
| SII | Mean FA in cerebral peduncle on FA skeleton (left) | 0.000 | 0.001 |
| SII | Mean FA in cerebral peduncle on FA skeleton (right) | 0.000 | 0.006 |
| SII | Mean FA in external capsule on FA skeleton (left) | 0.000 | 0.012 |
| SII | Mean FA in posterior limb of internal capsule on FA skeleton (right) | 0.000 | 0.032 |
| SII | Mean FA in retrolenticular part of internal capsule on FA skeleton (right) | 0.000 | 0.040 |
| SII | Mean FA in superior fronto-occipital fasciculus on FA skeleton (left) | 0.000 | 0.037 |
| SII | Mean FA in tapetum on FA skeleton (left) | 0.000 | 0.020 |

Abbreviations: CRP, C-reactive protein; PLR, platelets/lymphocytes ratio; SII, systemic immune-inflammation index (neutrophils×platelets/lymphocytes).

**Table S7.** Associations between peripheral immunity markers and MD (mean diffusivity) values of white matter tracts

| **Predictor** | **Outcome** | **ES** | **Adjusted_p** |
| --- | --- | --- | --- |
| CRP | Mean MD in pontine crossing tract on FA skeleton | -0.011 | 0.001 |
| Monocyte | Mean MD in anterior limb of internal capsule on FA skeleton (left) | 0.199 | 0.001 |
| Monocyte | Mean MD in anterior limb of internal capsule on FA skeleton (right) | 0.171 | 0.016 |
| Monocyte | Mean MD in external capsule on FA skeleton (right) | 0.193 | 0.002 |
| Neutrophil | Mean MD in anterior corona radiata on FA skeleton (left) | 0.030 | 0.006 |
| Neutrophil | Mean MD in anterior corona radiata on FA skeleton (right) | 0.030 | 0.007 |
| Neutrophil | Mean MD in genu of corpus callosum on FA skeleton | 0.030 | 0.007 |
| Neutrophil | Mean MD in superior corona radiata on FA skeleton (left) | 0.029 | 0.010 |
| Neutrophil | Mean MD in superior fronto-occipital fasciculus on FA skeleton (left) | 0.035 | 0.000 |
| NLR | Mean MD in anterior corona radiata on FA skeleton (left) | 0.033 | 0.046 |
| NLR | Mean MD in cingulum cingulate gyrus on FA skeleton (left) | 0.038 | 0.011 |
| NLR | Mean MD in cingulum cingulate gyrus on FA skeleton (right) | 0.038 | 0.011 |
| NLR | Mean MD in external capsule on FA skeleton (left) | 0.034 | 0.028 |
| NLR | Mean MD in retrolenticular part of internal capsule on FA skeleton (right) | 0.039 | 0.005 |
| NLR | Mean MD in superior longitudinal fasciculus on FA skeleton (right) | 0.033 | 0.042 |
| PLR | Mean MD in anterior corona radiata on FA skeleton (left) | 0.001 | 0.048 |
| PLR | Mean MD in anterior limb of internal capsule on FA skeleton (left) | 0.001 | 0.034 |
| PLR | Mean MD in cerebral peduncle on FA skeleton (left) | 0.001 | 0.000 |
| PLR | Mean MD in cerebral peduncle on FA skeleton (right) | 0.001 | 0.049 |
| PLR | Mean MD in cingulum cingulate gyrus on FA skeleton (left) | 0.001 | 0.002 |
| PLR | Mean MD in cingulum cingulate gyrus on FA skeleton (right) | 0.001 | 0.007 |
| PLR | Mean MD in cingulum hippocampus on FA skeleton (left) | 0.001 | 0.010 |
| PLR | Mean MD in cingulum hippocampus on FA skeleton (right) | 0.001 | 0.019 |
| PLR | Mean MD in external capsule on FA skeleton (left) | 0.001 | 0.008 |
| PLR | Mean MD in external capsule on FA skeleton (right) | 0.001 | 0.038 |
| PLR | Mean MD in posterior limb of internal capsule on FA skeleton (right) | 0.001 | 0.009 |
| PLR | Mean MD in posterior thalamic radiation on FA skeleton (right) | 0.001 | 0.024 |
| PLR | Mean MD in retrolenticular part of internal capsule on FA skeleton (left) | 0.001 | 0.046 |
| PLR | Mean MD in retrolenticular part of internal capsule on FA skeleton (right) | 0.001 | 0.001 |
| PLR | Mean MD in sagittal stratum on FA skeleton (left) | 0.001 | 0.002 |
| PLR | Mean MD in sagittal stratum on FA skeleton (right) | 0.001 | 0.001 |
| PLR | Mean MD in superior longitudinal fasciculus on FA skeleton (right) | 0.001 | 0.045 |
| Platelet | Mean MD in anterior corona radiata on FA skeleton (left) | 0.001 | 0.004 |
| Platelet | Mean MD in anterior corona radiata on FA skeleton (right) | 0.001 | 0.033 |
| Platelet | Mean MD in anterior limb of internal capsule on FA skeleton (left) | 0.001 | 0.010 |
| Platelet | Mean MD in genu of corpus callosum on FA skeleton | 0.001 | 0.000 |
| Platelet | Mean MD in posterior limb of internal capsule on FA skeleton (left) | 0.001 | 0.002 |
| Platelet | Mean MD in posterior limb of internal capsule on FA skeleton (right) | 0.001 | 0.026 |
| Platelet | Mean MD in retrolenticular part of internal capsule on FA skeleton (left) | 0.001 | 0.035 |
| Platelet | Mean MD in superior corona radiata on FA skeleton (right) | 0.001 | 0.043 |
| Platelet | Mean MD in superior fronto-occipital fasciculus on FA skeleton (left) | 0.001 | 0.027 |
| SII | Mean MD in anterior corona radiata on FA skeleton (left) | 0.000 | 0.000 |
| SII | Mean MD in anterior corona radiata on FA skeleton (right) | 0.000 | 0.001 |
| SII | Mean MD in anterior limb of internal capsule on FA skeleton (left) | 0.000 | 0.003 |
| SII | Mean MD in anterior limb of internal capsule on FA skeleton (right) | 0.000 | 0.012 |
| SII | Mean MD in cingulum cingulate gyrus on FA skeleton (left) | 0.000 | 0.000 |
| SII | Mean MD in cingulum cingulate gyrus on FA skeleton (right) | 0.000 | 0.000 |
| SII | Mean MD in cingulum hippocampus on FA skeleton (left) | 0.000 | 0.030 |
| SII | Mean MD in external capsule on FA skeleton (left) | 0.000 | 0.001 |
| SII | Mean MD in external capsule on FA skeleton (right) | 0.000 | 0.011 |
| SII | Mean MD in genu of corpus callosum on FA skeleton | 0.000 | 0.000 |
| SII | Mean MD in posterior limb of internal capsule on FA skeleton (right) | 0.000 | 0.003 |
| SII | Mean MD in retrolenticular part of internal capsule on FA skeleton (left) | 0.000 | 0.005 |
| SII | Mean MD in retrolenticular part of internal capsule on FA skeleton (right) | 0.000 | 0.000 |
| SII | Mean MD in splenium of corpus callosum on FA skeleton | 0.000 | 0.024 |
| SII | Mean MD in superior corona radiata on FA skeleton (left) | 0.000 | 0.001 |
| SII | Mean MD in superior corona radiata on FA skeleton (right) | 0.000 | 0.007 |
| SII | Mean MD in superior fronto-occipital fasciculus on FA skeleton (left) | 0.000 | 0.018 |
| SII | Mean MD in superior longitudinal fasciculus on FA skeleton (left) | 0.000 | 0.006 |
| SII | Mean MD in superior longitudinal fasciculus on FA skeleton (right) | 0.000 | 0.001 |
| SII | Mean MD in tapetum on FA skeleton (left) | 0.000 | 0.013 |

Abbreviations: CRP, C-reactive protein; NLR, neutrophils/lymphocytes ratio; PLR, platelets/lymphocytes ratio; SII, systemic immune-inflammation index (neutrophils×platelets/lymphocytes).

**Table S8.**  Associations between peripheral immunity markers and the volume of cortical and subcortical regions

| **Predictor** | **Outcome** | **ES** | **Adjusted_p** |
| --- | --- | --- | --- |
| CRP | BrainSegVol_to_eTIV | -0.011 | 0.001 |
| CRP | Right_choroid_plexus | 0.008 | 0.029 |
| Monocyte | CerebralWhiteMatterVol | -0.166 | 0.002 |
| Monocyte | lhCerebralWhiteMatterVol | -0.166 | 0.002 |
| Monocyte | rhCerebralWhiteMatterVol | -0.165 | 0.002 |
| Monocyte | Right_VentralDC | -0.168 | 0.002 |
| Monocyte | BrainSegVolNotVent | -0.148 | 0.007 |
| Monocyte | BrainSegVol | -0.145 | 0.008 |
| Monocyte | BrainSegVolNotVentSurf | -0.146 | 0.008 |
| Monocyte | SupraTentorialVolNotVentVox | -0.140 | 0.020 |
| Monocyte | SupraTentorialVolNotVent | -0.138 | 0.025 |
| Monocyte | SupraTentorialVol | -0.136 | 0.027 |
| Monocyte | Left_Cerebellum_Cortex | -0.142 | 0.045 |
| Neutrophil | BrainSegVol_to_eTIV | -0.047 | 0.000 |
| Neutrophil | CortexVol | -0.037 | 0.000 |
| Neutrophil | lhCortexVol | -0.036 | 0.000 |
| Neutrophil | TotalGrayVol | -0.035 | 0.000 |
| Neutrophil | rhCortexVol | -0.036 | 0.000 |
| Neutrophil | Left_insula | -0.037 | 0.000 |
| Neutrophil | BrainSegVolNotVent | -0.033 | 0.000 |
| Neutrophil | SupraTentorialVolNotVentVox | -0.033 | 0.000 |
| Neutrophil | Right_medialorbitofrontal | -0.037 | 0.000 |
| Neutrophil | BrainSegVolNotVentSurf | -0.033 | 0.000 |
| Neutrophil | SurfaceHoles | 0.038 | 0.000 |
| Neutrophil | SupraTentorialVolNotVent | -0.033 | 0.000 |
| Neutrophil | Right_insula | -0.034 | 0.000 |
| Neutrophil | rhSurfaceHoles | 0.037 | 0.000 |
| Neutrophil | lhSurfaceHoles | 0.036 | 0.000 |
| Neutrophil | Right_superiortemporal | -0.034 | 0.000 |
| Neutrophil | Left_superiorfrontal | -0.033 | 0.000 |
| Neutrophil | Left_parstriangularis | -0.034 | 0.000 |
| Neutrophil | BrainSegVol | -0.028 | 0.000 |
| Neutrophil | Left_precentral | -0.033 | 0.000 |
| Neutrophil | Left_medialorbitofrontal | -0.032 | 0.001 |
| Neutrophil | Left_parsorbitalis | -0.033 | 0.001 |
| Neutrophil | SupraTentorialVol | -0.028 | 0.001 |
| Neutrophil | Right_posteriorcingulate | -0.033 | 0.001 |
| Neutrophil | Right_Inf_Lat_Vent | 0.031 | 0.001 |
| Neutrophil | Left_lateralorbitofrontal | -0.030 | 0.002 |
| Neutrophil | Right_lateralorbitofrontal | -0.030 | 0.002 |
| Neutrophil | Left_paracentral | -0.032 | 0.002 |
| Neutrophil | Left_superiortemporal | -0.029 | 0.003 |
| Neutrophil | Right_inferiortemporal | -0.029 | 0.003 |
| Neutrophil | lhCerebralWhiteMatterVol | -0.027 | 0.003 |
| Neutrophil | Right_lateraloccipital | -0.028 | 0.005 |
| Neutrophil | Left_fusiform | -0.029 | 0.005 |
| Neutrophil | CerebralWhiteMatterVol | -0.027 | 0.005 |
| Neutrophil | Right_parsopercularis | -0.030 | 0.006 |
| Neutrophil | Right_inferiorparietal | -0.028 | 0.006 |
| Neutrophil | Left_parsopercularis | -0.030 | 0.006 |
| Neutrophil | Left_entorhinal | -0.030 | 0.006 |
| Neutrophil | Right_precentral | -0.029 | 0.006 |
| Neutrophil | Right_superiorfrontal | -0.028 | 0.006 |
| Neutrophil | Right_paracentral | -0.029 | 0.007 |
| Neutrophil | Right_fusiform | -0.028 | 0.007 |
| Neutrophil | rhCerebralWhiteMatterVol | -0.026 | 0.008 |
| Neutrophil | Right_rostralmiddlefrontal | -0.027 | 0.009 |
| Neutrophil | Right_Accumbens_area | -0.028 | 0.009 |
| Neutrophil | Right_caudalmiddlefrontal | -0.029 | 0.012 |
| Neutrophil | Right_parstriangularis | -0.027 | 0.022 |
| Neutrophil | Right_entorhinal | -0.028 | 0.024 |
| Neutrophil | Left_precuneus | -0.026 | 0.025 |
| Neutrophil | Left_middletemporal | -0.025 | 0.027 |
| Neutrophil | Right_parsorbitalis | -0.026 | 0.030 |
| Neutrophil | Left_postcentral | -0.026 | 0.030 |
| Neutrophil | Left_posteriorcingulate | -0.027 | 0.034 |
| Neutrophil | Left_inferiorparietal | -0.026 | 0.036 |
| Neutrophil | Left_rostralmiddlefrontal | -0.025 | 0.039 |
| Neutrophil | Third_Ventricle | 0.024 | 0.044 |
| Platelet | Left_posteriorcingulate | -0.001 | 0.034 |
| NLR | Right_parstriangularis | -0.035 | 0.010 |
| NLR | Right_parsopercularis | -0.034 | 0.025 |
| NLR | BrainSegVol_to_eTIV | -0.033 | 0.040 |
| SII | Right_parstriangularis | 0.000 | 0.006 |
| SII | Left_parsorbitalis | 0.000 | 0.016 |
| SII | Left_posteriorcingulate | 0.000 | 0.020 |
| SII | Left_paracentral | 0.000 | 0.027 |
| SII | Left_Inf_Lat_Vent | 0.000 | 0.027 |
| SII | Right_parsorbitalis | 0.000 | 0.029 |
| SII | Right_parsopercularis | 0.000 | 0.029 |
| SII | Right_Accumbens_area | 0.000 | 0.035 |
| SII | Right_posteriorcingulate | 0.000 | 0.047 |

Abbreviations: CRP, C-reactive protein; NLR, neutrophils/lymphocytes ratio; PLR, platelets/lymphocytes ratio; SII, systemic immune-inflammation index (neutrophils×platelets/lymphocytes).

**Table S9.**  Associations between peripheral immunity markers and the area of cortical regions

| **Predictor** | **Outcome** | **ES** | **Adjusted_p** |
| --- | --- | --- | --- |
| Monocyte | Left_cuneus | -0.162 | 0.013 |
| Neutrophil | Right_medialorbitofrontal | -0.032 | 0.000 |
| Neutrophil | Left_parsorbitalis | -0.032 | 0.000 |
| Neutrophil | Left_lateralorbitofrontal | -0.031 | 0.000 |
| Neutrophil | Right_posteriorcingulate | -0.031 | 0.001 |
| Neutrophil | Left_parstriangularis | -0.030 | 0.001 |
| Neutrophil | Right_parsorbitalis | -0.025 | 0.016 |
| Neutrophil | Right_superiortemporal | -0.025 | 0.019 |
| Neutrophil | Right_rostralmiddlefrontal | -0.025 | 0.019 |
| Neutrophil | Left_entorhinal | -0.026 | 0.030 |
| Neutrophil | Left_superiortemporal | -0.023 | 0.038 |
| Neutrophil | Right_lateralorbitofrontal | -0.024 | 0.045 |
| NLR | Right_parstriangularis | -0.035 | 0.007 |
| SII | Left_parsorbitalis | 0.000 | 0.016 |
| SII | Right_parstriangularis | 0.000 | 0.023 |
| SII | Left_parstriangularis | 0.000 | 0.029 |

Abbreviations: NLR, neutrophils/lymphocytes ratio; SII, systemic immune-inflammation index (neutrophils×platelets/lymphocytes).

**Table S10.**  Associations between peripheral immunity markers and the thickness of cortical regions

| **Predictor** | **Outcome** | **ES** | **Adjusted_p** |
| --- | --- | --- | --- |
| Monocyte | Left_cuneus | 0.157 | 0.038 |
| Monocyte | Left_pericalcarine | 0.156 | 0.045 |
| Neutrophil | Right_precentral | -0.035198646 | 0.000170733 |
| Neutrophil | Right_fusiform | -0.032921911 | 0.000878871 |
| Neutrophil | Left_precentral | -0.032494032 | 0.000945858 |
| Neutrophil | Right_paracentral | -0.030615545 | 0.003684782 |
| Neutrophil | Left_superiorfrontal | -0.029320178 | 0.005112505 |
| Neutrophil | Left_paracentral | -0.027835372 | 0.015901514 |
| Neutrophil | Right_inferiortemporal | -0.027842473 | 0.019185991 |
| Neutrophil | Right_superiortemporal | -0.026386594 | 0.028515813 |
| CRP | Left_rostralanteriorcingulate | -0.009 | 0.016 |
| CRP | Left_paracentral | -0.009 | 0.030 |
| NLR | Left_precentral | -0.032 | 0.037 |
| PLR | Left_frontalpole | -0.001 | 0.022 |
| SII | Left_precentral | 0.000 | 0.028 |

Abbreviations: CRP, C-reactive protein; NLR, neutrophils/lymphocytes ratio; PLR, platelets/lymphocytes ratio; SII, systemic immune-inflammation index (neutrophils×platelets/lymphocytes).
